# Supplementary material for: 177Lu-DOTATATE Efficacy and Safety in Functioning Neuroendocrine Tumors: A Joint Analysis of Phase II Prospective Clinical Trials
Source: Cancers (Basel). 2022 Dec 7;14(24):6022. doi: 10.3390/cancers14246022 (PMC9776442; doi:10.3390/cancers14246022)
Supplement: Supplementary file 1 [file cancers-14-06022-s001.zip › cancers-1937393-supplementary.docx]

Supplementary Materials

**^177^Lu-DOTATATE Efficacy and Safety in Functioning Neuroendocrine Tumors: A Joint Analysis of Phase II Prospective Clinical Trials**

**Alberto Bongiovanni, Silvia Nicolini, Toni Ibrahim, Flavia Foca, Maddalena Sansovini, Arianna Di Paolo, Ilaria Grassi, Chiara Liverani, Chiara Calabrese, Nicoletta Ranallo, Federica Matteucci, Giovanni Paganelli and Stefano Severi**

**Table S1.** Risk factors for renal injury and comorbidities.

|  | **No. (%)** |
| --- | --- |
| Presence of risk factors |  |
| No | 39 (57.3) |
| Previous cancer treatment | 15 (22.0) |
| Hypertension | 4 (6.0) |
| Other risk factors | 10 (14.7) |
| Presence of cardiovascular comorbidities | 39 (57.3) |
| Hypertension | 30 (76.9) |
| Other cardiovascular comorbidities | 9 (23.1) |
| Presence of metabolic/endocrine comorbidities | 20 (29.4) |
| Diabetes | 10 (50.0) |
| Other metabolic/endocrine comorbidities | 10 (50.0) |

**Table S2.** Univariate analysis of clinical factors for OS.

| **Variables** |  | **No. patients** | **No. events** | **2-year OS**  **(95%CI)** | ***P*-value (log-rank test)** |
| --- | --- | --- | --- | --- | --- |
| Total | | 68 | 11 | 87.8 (76.1−94.1) | - |
| Age at treatment, years | <65 | 32 | 3 | 92.8 (74.0−98.1) | 0.204 |
|  | ≥65 | 36 | 8 | 83.9 (65.2−93.0) |  |
| Gender | Male | 38 | 6 | 91.3 (75.5−97.1) | 0.998 |
|  | Female | 30 | 5 | 84.4 (63.6−93.9) |  |
| Site of disease | Gastrointestinal | 55 | 9 | 87.0 (73.3−94.0) | 0.821 |
|  | Lung | 9 | 2 | 87.5 (38.7−98.1) |  |
|  | Pancreas | 1 | 0 | - |  |
|  | Other | 3 | 0 | - |  |
| Presence of metastasis at diagnosis | Yes | 51 | 8 | 86.0 (71.3−93.5) | 0.569 |
|  | No | 17 | 3 | 93.7 (63.2−99.1) |  |
| Previous surgery | Yes | 43 | 5 | 94.1 (78.4−98.4) | 0.049 |
|  | No | 25 | 6 | 76.1 (51.3−89.4) |  |
| ECOG PS | 0 | 52 | 4 | 95.6 (83.5−98.8) | 0.014 |
|  | 1 | 15 | 6 | 70.7 (39.3−87.9) |  |
|  | 2 | 1 | 1 | - |  |
| Ki-67 | Ki-67 ≤2 | 20 | 3 | 94.1 (65.0−99.1) | 0.305 |
|  | Ki-67 >2 and ≤20 | 45 | 7 | 86.8 (71.0−94.3) |  |
|  | Ki-67 >20 | 2 | 1 | - |  |
| Ki-67 | Ki-67 ≤10 | 50 | 6 | 92.6 (78.5−97.6) | 0.195 |
|  | Ki-67 >10 | 17 | 5 | 75 (46.8−89.9) |  |
| Grading | G1 | 18 | 2 | 93.7 (63.2−99.1) | 0.420 |
|  | G2 | 48 | 8 | 87.4 (72.1−94.6) |  |
|  | G3 | 2 | 1 | - |  |
| Carcinoid heart disease | Yes | 5 | 1 | 80.0 (20.3−96.9) | - |
|  | No | 60 | 10 | 88.1 (75.4−94.5) |  |
| ^18^F-FDG PET/CT | Posititve | 26 | 6 | 87.3 (65.5−95.7) | 0.159 |
|  | Negative | 28 | 3 | 87.1 (64.9−95.6) |  |

OS, overall survival; ECOG PS, Eastern Cooperative Oncology Group performance status.

**Table S3.** Univariate analysis of clinical factors for PFS.

| **Variables** | | **No. patients** | **No. events** | **Median PFS (95% CI)** | **2-year PFS (95% CI)** | ***P*-value**  **(log-rank test)** |
| --- | --- | --- | --- | --- | --- | --- |
| Total | | 66 | 30 | 33.0 (27.1−48.2) | 74.7 (60.7−84.3) | - |
| Age at treatment, years | <65 | 31 | 13 | 38.7 (25.2−NE) | 77.9 (57.1−89.4) | 0.421 |
|  | ≥65 | 35 | 17 | 31.2 (26.2−48.2) | 71.6 (50.4−84.9) |  |
| Gender | Male | 37 | 15 | 39.5 (26.2−50.9) | 80.8 (61.7−91.0) | 0.585 |
|  | Female | 29 | 15 | 31.2 (21.3−38.7) | 68.1 (45.6−82.8) |  |
|  | Other | 3 | 1 | - | - |  |
| Presence of metastasis at diagnosis | Yes | 49 | 23 | 38.7 (25.2−50.8) | 70.6 (53.8−82.2) | 0.432 |
|  | No | 17 | 7 | 30.7 (26.2−NE) | 88.2 (60.6−96.9) |  |
| Previous surgery | Yes | 43 | 17 | 39.2 (28.3−54.1) | 82.9 (65.6−92.1) | 0.027 |
|  | No | 23 | 13 | 24.9 (13.8−39.5) | 57.9 (32.3−76.8) |  |
| ECOG PS | 0 | 51 | 21 | 38.7 (28.3−54.1) | 80.1 (63.8−89.7) | 0.040 |
|  | 1 | 14 | 8 | 25.2 (11.4−NE) | 61.9 (30.7−82.2) |  |
|  | 2 | 1 | 1 | - | - |  |
| Ki-67 | Ki-67 ≤2 | 19 | 8 | 39.2 (19.7−NE) | 73.3 (43.3−89.1) | 0.515 |
|  | Ki-67 >2 and ≤20 | 44 | 20 | 33.1 (27.1−48.2) | 78.5 (61.1−88.8) |  |
|  | Ki-67 >20 | 2 | 2 | - | - |  |
| Ki-67 | Ki-67 ≤10 | 48 | 17 | 39.5 (28.3−NE) | 82.1 (65.7−91.1) | 0.002 |
|  | Ki-67 >10 | 17 | 13 | 25.3 (11.4−31.2) | 54.6 (27.1−75.5) |  |
| Grading | G1 | 18 | 6 | 54.1 (19.7−NE) | 78.6 (47.0−92.7) | 0.106 |
|  | G2 | 46 | 22 | 31.2 (26.2−39.5) | 77.1 (60.2−87.5) |  |
|  | G3 | 2 | 2 | - | - |  |
| Carcinoid heart disease | Yes | 5 | 2 | 33.1 (2.2−NE) | 80.0 (20.4−96.9) | 0.851 |
|  | No | 58 | 25 | 38.7 (26.2−50.8) | 75.1 (59.8−85.1) |  |
| ^18^F-FDG PET/CT | Positive | 26 | 12 | 38.7 (17.1−NE) | 63.5 (39.7−79.9) | 0.076 |
|  | Negative | 28 | 10 | 54.1 (27.1−NE) | 86.4 (63.1−95.5) |  |

PFS, progression-free survival; ECOG PS, Eastern Cooperative Oncology Group performance status; NE, not estimable.

**Table S4.** Comparison between median time to disease response and syndrome.

| **Variables** | **No. patients (%)** | **No. events** | **Median time (95% CI)** |
| --- | --- | --- | --- |
| Time to best response | 64 | 64 | 7.3 (5.8−7.9) |
| Time to response to syndrome | 66 | 58 | 5.0 (4.0−6.5) |


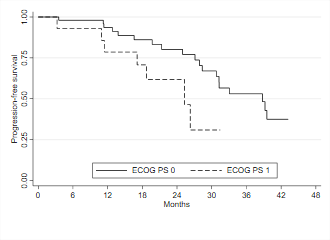


**Figure S1.** PFS curves according to ECOG PS 0 *vs.* 1.


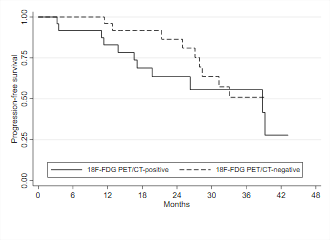


**Figure S2.** PFS curves according to ^18^FDG-PET/CT.


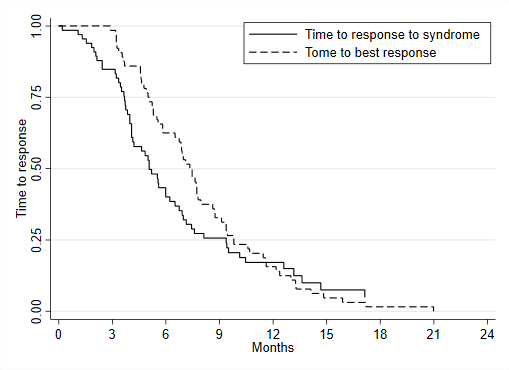


**Figure S3.** Difference in time to syndrome response and time to best response.


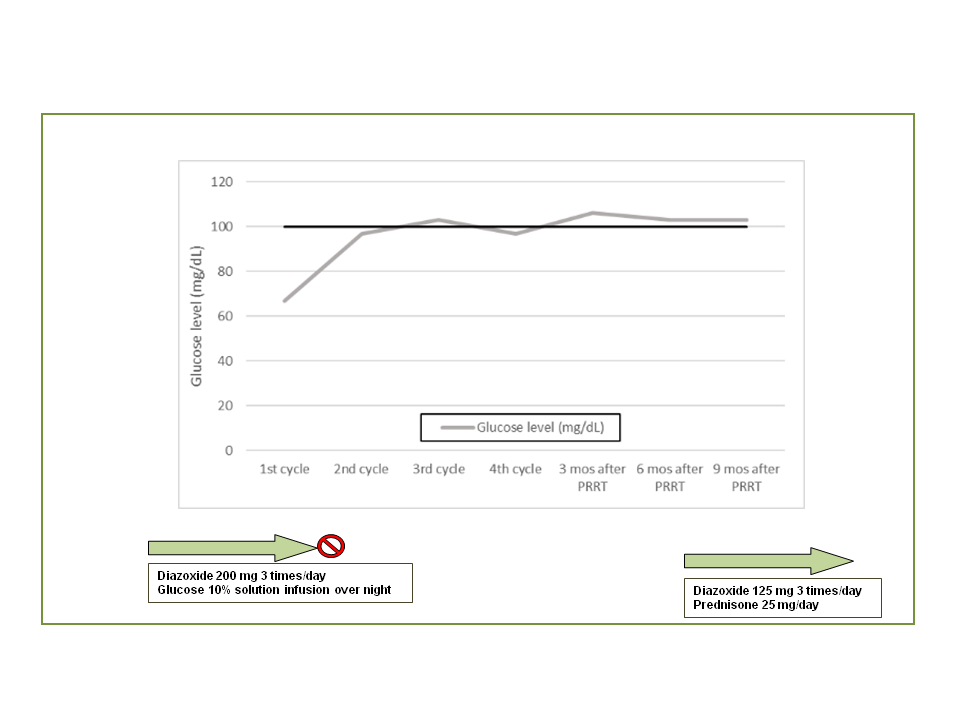


**Figure S4.** Glucose levels of a patient with malignant insulinoma undergoing ^177^Lu-PRRT, with a focus on supportive therapy received.
